# Qualitative Properties of Circulating Fatty Acids Are Associated With MASLD: A Cross‐Sectional Study From the NHANES Database

**DOI:** 10.1111/liv.70441

**Published:** 2025-11-14

**Authors:** Stefano Ciardullo, Michela Vergani, Mariangela Rizzo, Alice Oltolini, Alessia Bongo, Emanuele Muraca, Gianluca Perseghin

**Affiliations:** ^1^ Department of Medicine and Rehabilitation Policlinico di Monza Monza Italy; ^2^ Department of Medicine and Surgery University of Milano Bicocca Milan Italy

**Keywords:** FFAs, MASH, MASLD, PUFAs

## Abstract

**Background and Objective:**

The role of circulating fatty acids (FA) as well as the association between their composition and metabolic dysfunction‐associated steatotic liver disease (MASLD) is not known. Here, we evaluate the association between specific FA subtypes and both MASLD and liver fibrosis in a general population setting.

**Methods:**

In this cross‐sectional study, serum levels of FAs were measured after an overnight fast in a representative sample of the general US population. Concentrations of total FA were assessed using capillary gas chromatography coupled with negative‐ion mass spectrometry. They were divided into saturated (palmitic, stearic and butyric acid), monounsaturated (palmitoleic and oleic acid), ω‐6 polyunsaturated (ω‐6 PUFA: linoleic and arachidonic acid) and ω‐3 PUFA (alpha‐linolenic, docosanoic [DHA] and eicosapentaenoic [EPA] acid). Explorative end points were: insulin resistance, liver steatosis and fibrosis estimated using the Homeostatic Model of Insulin Resistance (HOMA‐IR), a fatty liver index (FLI) score ≥ 60 and a Fibrosis 4 (FIB‐4) score ≥ 1.3, respectively. Logistic regression analyses were performed to assess their associations with different FAs after adjustment for confounders.

**Results:**

A total of 2440 people were included in the study. Concentrations of all considered FAs increased progressively across HOMA‐IR quartiles. A similar trend was identified when the population was segregated according to FLI. In logistic regression analyses adjusted for age, sex, body mass index and race‐ethnicity, when evaluated as a percentage of all circulating FAs, linoleic acid, arachidonic acid and DHA were inversely associated with insulin resistance and liver steatosis. Finally, only alpha‐linolenic acid was associated with a lower risk of liver fibrosis (OR: 0.48, 95% CI 0.25–0.93, *p* = 0.031).

**Conclusions:**

This study provides initial epidemiologic evidence in humans that ω‐3 PUFA in particular are associated with a lower risk of liver steatosis and alpha‐linolenic acid in particular was also protective against fibrosis, supporting data in animal models. Moreover, their circulating levels might be manipulated using nutritional strategies in clinical studies.

**Abbreviations**
DHA: docosahexaenoic acid
EPA: eicosapentaenoic acid
FLI: fatty liver index
MASLD: metabolic dysfunction‐associated steatotic liver disease
NAFLD: non‐alcoholic fatty liver disease
NHANES: National Health and Nutrition Examination Survey

**Summary**

- In this study, we evaluated the association between circulating fatty acids and both MASLD and liver fibrosis in the general US population.
- We show that not only the total concentration of FAs, but also their composition affects the risk of liver steatosis and fibrosis, opening the possibility of modifying liver disease by specific FA supplementation.

## 1 Introduction

Metabolic dysfunction‐associated steatotic liver disease (MASLD) [1], previously known as non‐alcoholic fatty liver disease (NAFLD), represents the most commonly encountered chronic liver condition in clinical practice. Recent estimates identified a prevalence of approximately 30% in the general adult population, with varying rates around the globe based on ethnicity and other risk factors [2]. The pathogenesis of MASLD is strictly connected with the metabolic syndrome and its components including hypertension [3], prediabetes and type 2 diabetes [4], central obesity [5] and dyslipidaemia. Indeed, some authors still consider MASLD as the hepatic manifestation of insulin resistance [6]. Circulating fatty acids (FA) levels are associated with the insulin resistance syndrome [7, 8] and with MASLD [9] and their higher circulating levels are considered the manifestation of the lack of insulin control on the lipolytic pathway at the level of the adipose tissue. While the deleterious metabolic impact of the excessive flux of FA, especially in the fasting state, from the adipose tissue to the skeletal muscle [10] and to the liver [11] is well recognised, the supply of FA to the liver comes also from other sources [12]. In particular, in the fed state, FA can reach the liver in their esterified form as part of chylomicron remnant particles containing triglycerides, or as free FA as a result of spillover.

From a structural perspective, FAs are classified as either saturated or unsaturated, based on whether they lack or contain a carbon‐to‐carbon double bond [13]. Unsaturated fatty acids are further categorised into two types: monounsaturated fatty acids, which have a single double bond, and polyunsaturated fatty acids (PUFAs), which have two or more double bonds. Common monounsaturated fatty acids include palmitoleic and oleic acids. PUFAs are divided into two families based on the original fatty acids they are derived from: n‐6 (omega‐6) PUFAs, originating from linoleic acid, and n‐3 (omega‐3) PUFAs, derived from alpha‐linolenic acid [14]. In a recent study performed on the UK Biobank, plasma saturated FAs and monounsaturated FAs were associated with a higher risk of incident severe NAFLD, whereas plasma n‐3 PUFAs, n‐6 PUFAs and linoleic acid were associated with a lower risk [15]. While the association between overall circulating free FA levels as well as triglycerides and MASLD has been studied, data are scarce on the impact of total FA levels, as well as of properties on MASLD development and progression [16]. Indeed a large part of total circulating FAs is esterified in cholesterol esters and phospholipids.

In fact, while the absolute risk of developing cirrhosis or hepatocellular carcinoma is low in the whole MASLD population [17], it increases substantially in patients with steato‐hepatitis (MASH) and particularly with worsening of liver fibrosis [18, 19]. These aspects, along with its high prevalence made it one of the most common causes of liver‐related events in recent years [20, 21]. Previous studies focusing on the association between FAs and liver endpoints focused mostly on free FAs and were conducted in single research hospital settings, while data from population‐based studies are scarce.

In this context, in the present population‐based cross‐sectional study we used data from the National Health and Nutrition Examination Survey (NHANES) to study the association between circulating levels of different FAs and MASLD and related liver fibrosis in the general US population.

## 2 Materials and Methods

This study represents an analysis of data obtained during the 2011–2014 cycles of the NHANES, a complex cross‐sectional survey programme conducted in the United States by the National Center for Health Statistics of the Centers for Disease Control and Prevention. NHANES aims to include a representative sample of the general, non‐institutionalised US population of all ages. To this end, it applies a stratified, multistage, clustered probability sampling design. As with previous NHANES samples, a four‐stage sample design was used in NHANES 2011–2014. More information on sampling methods is available from the NHANES sample design document [22].

In order to obtain enough data on minorities for statistical analysis, oversampling of non‐Hispanic Black and Hispanic persons, people with low income and older adults is performed. The survey consists of two main parts: a structured interview conducted in the participants' home and a standardised health examination performed at a mobile examination center (MEC), which includes a physical examination as well as laboratory and imaging tests. Full methodology of data collection is available on the NHANES website [23]. The original survey was approved by the Centers for Disease Control and Prevention Research Ethics Review Board and written informed consent was obtained from all adult participants. The present analysis was deemed exempt by the Institutional Review Board at our institution, as the dataset used in the analysis was completely de‐identified.

### 2.1 Laboratory Tests and Clinical Data

Participants self‐reported age, sex, race‐ethnicity (categorised as non‐Hispanic White, non‐Hispanic Black, Hispanic or other), education, smoking status and previous medical history. Body measurements including height (cm), weight (kg) and waist circumference (cm) were ascertained during the MEC visit; body mass index (BMI) was calculated as weight in kilograms divided by height in metres squared. Diabetes was defined in accordance with the American Diabetes Association criteria if any of the following conditions were met: (1) A self‐reported diagnosis of diabetes; (2) use of anti‐diabetic drugs; (3) a haemoglobin A1c (HbA1c) level ≥ 6.5% (48 mmol/mol); (4) a fasting plasma glucose ≥ 126 mg/dL; (5) a random plasma glucose ≥ 200 mg/dL [24]. Laboratory methods for measurements of HbA1c and glucose are reported in detail elsewhere [25].

Hepatitis C virus infection was indicated by presence of viral RNA and/or a confirmed antibody test and hepatitis B virus infection as a positive surface antigen test, as described [26]. An immunometric technique is used. This involves the simultaneous reaction of HBsAg in the sample with mouse monoclonal anti‐HBs antibody coated onto the wells and a horseradish peroxidase (HRP)‐labelled mouse monoclonal anti‐HBs antibody in the conjugate.

**FIGURE 1:**
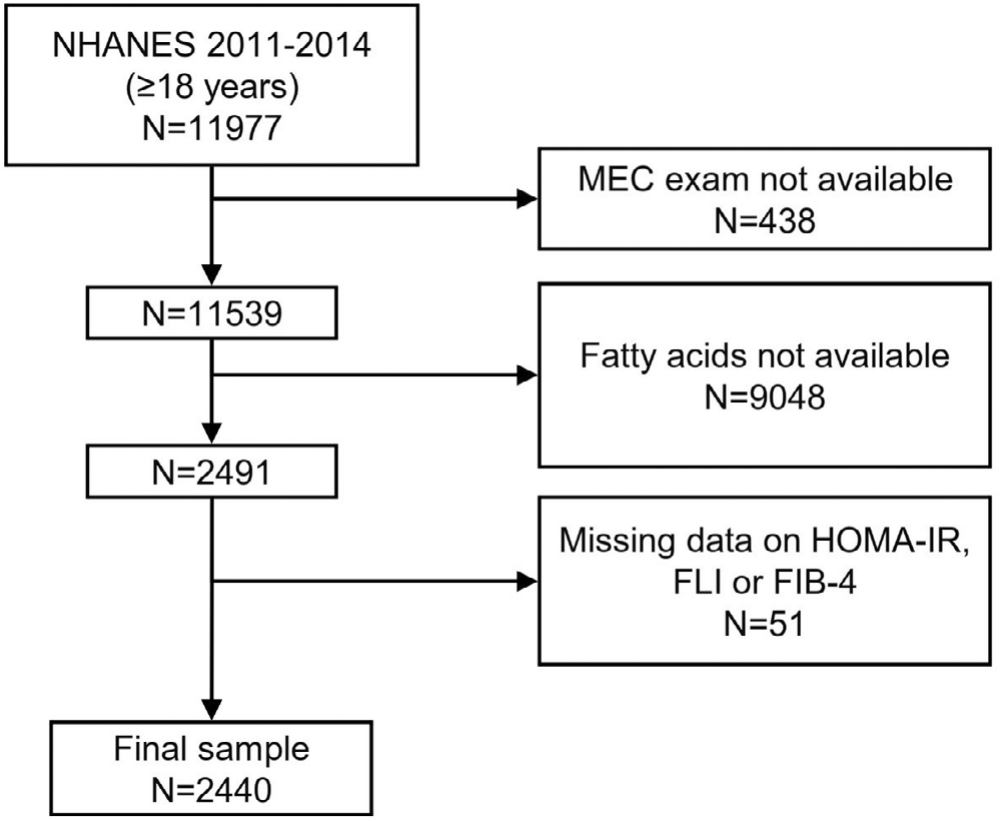
Flow chart of the study participants. MEC, mobile examination center; NHANES, National Health and Nutrition Examination Survery.

Alcohol consumption was estimated based on self‐reported data on the amount and frequency of alcohol use within the previous year. It was considered significant if > 1 drink per day for women and > 2 drinks per day for men on average [27]. We excluded participants that reported significant alcohol consumption or that tested positive for current chronic viral hepatitis B or C (Figure 1).

### 2.2 Evaluation of Circulating FA Levels

Data on circulating FAs are available from the 2011–2012 and 2013–2014 NHANES cycles. The original goal of this evaluation was to obtain US reference ranges for most circulating FAs. The applied assay works as follows. Esterified FAs are released from triglycerides, phospholipids and cholesteryl esters by treating them sequentially with mineral acid and base, along with heat. Based on a modified method from Lagerstedt et al. [28], fatty acids are extracted from a sample matrix (100 μL serum or plasma) using hexane. An internal standard solution, containing 18 stable isotopically labelled FAs, is added to ensure accurate recovery. This extracted mixture is then derivatised using pentafluorobenzyl bromide and triethylamine, producing pentafluorobenzyl esters. The resulting mixture is analysed using a capillary gas chromatograph column to separate the target FAs from other components in the matrix. Detection of the FAs is carried out using electron capture negative‐ion mass spectrometry within a 34‐min timeframe. A total of 30 FAs, comprising 11 saturated, six monounsaturated and 13 PUFAs, are quantified using selected ion monitoring. Quantification involves comparing the peak area of the FAs in the unknown sample to that of a known amount in a calibration solution. Corrections are made by comparing the peak area of the internal standard in the unknown sample to its counterpart in the calibration solution. For a comprehensive explanation of the laboratory methods employed, refer to the NHANES Laboratory Method Files section [29]. We focused the present analysis on the following molecules: saturated (palmitic, stearic and butyric acid), monounsaturated (palmitoleic and oleic acid), ω‐6 polyunsaturated (ω‐6 PUFA: linoleic and arachidonic acid) and ω‐3 PUFA (alpha‐linolenic, docosanoic [DHA] and eicosapentaenoic [EPA] acid).

### 2.3 Evaluation of Insulin Resistance, Liver Steatosis and Fibrosis

The degree of insulin resistance in the included population was estimated using the widely validated homeostatic model assessment of insulin resistance (HOMA‐IR), which is based on fasting insulin and fasting glucose concentrations [30]. A HOMA‐IR ≥ 2.5 was considered indicative of insulin resistance [31].

In the absence of liver biopsy data or imaging techniques, liver steatosis was estimated using the Fatty Liver Index (FLI). FLI was derived using the following formula:

$$\mathrm{FLI}:e^{y}/\left(1+e^{y}\right)\times 100$$

where *y* = 0.953 × ln(triglycerides, mg/dL) + 0.139 × BMI, kg/m^2^ + 0.718 × ln(gamma glutamyl transpeptidase, U/L) + 0.053 × waist circumference, cm – 15.745.

As originally proposed, a FLI ≥ 60 was considered indicative of liver steatosis. The score was validated compared with magnetic resonance spectroscopy in subsequent studies [32]. Risk of liver fibrosis was assessed using the Fibrosis‐4 Index (FIB‐4). This score was first developed by Sterling et al. [33] in patents with concomitant Hepatitis C virus (HCV) and human immunodeficiency virus (HIV) infection using liver biopsy as a gold standard reference. Its performance in the setting of NAFLD/MASLD was evaluated by Shah et al. [34] and by McPherson et al. [35], showing a good diagnostic performance with AUROCs of 0.80–0.86 to detect advanced liver fibrosis. The score is calculated according to the following formula [33]:

$$\begin{array}{c} \mathrm{FIB}‐4:\left(\mathrm{age}\;\left(\text{years}\right)\times \mathrm{AST}\;\left(\mathrm{IU}/L\right)\right)/\\ \left(\text{platelet count}\;(10^{9}/L)\times \mathrm{ALT}(\mathrm{IU}/L)\right) \end{array}$$

As recommended by international guidelines, a FIB‐4 ≥ 1.3 was considered as potentially indicative of liver fibrosis, as a valued below this threshold has a high negative predictive value [36, 37].

### 2.4 Statistical Analysis

All analyses were conducted using Stata version 16.0 (StataCorp, College Station, TX, USA), accounting for the complex survey design of NHANES. We used appropriate weighting for each analysis, as suggested by the NCHS. The weighting procedure is essential to derive accurate and representative results since the NHANES study oversamples some minorities based on age, ethnicity or other features. Data are expressed as weighted proportions ± standard error (SE) for categorical variables and as weighted means ± SE for continuous variables.

Participants' characteristics according to HOMA‐IR, liver steatosis and fibrosis status were compared using linear regression for continuous variables and the design‐adjusted Rao‐Scott chi‐square test for categorical variables. Multivariable logistic regression analysis was performed in order to evaluate the association of each FA with insulin resistance, MASLD and liver fibrosis. Covariates included in the model were age, sex, race‐ethnicity and BMI as a measure of overall fat mass. The choice of covariates followed a biological plausibility approach based on a large base of evidence showing that these are the major risk factors for liver steatosis and fibrosis. FAs levels were log‐transformed before including them in the logistic regression models. Given that we excluded participants with missing data on the main dependent and independent variables, missing values for all included variables affected < 1% of participants. We therefore excluded these participants from each specific analysis. A two‐tailed value of *p* < 0.05 was considered statistically significant.

## 3 Results

This study included 2440 participants older than 18 years with available data on circulating FAs, HOMA‐IR, FLI and FIB‐4. Features of the overall population according to BMI are shown in Table 1. Mean age was 46.2 years; 48.8% of participants were men and the mean BMI was 29.1 kg/m^2^. Most features differed significantly according to BMI, including concentrations of most FAs, except for EPA and DHA. Features of the overall population according to BMI are shown in Table 2. Participants with higher HOMA‐IR levels were more commonly men, showed higher BMI, SBP, triglycerides, HbA1c, ALTs and FLI; they also had a higher prevalence of diabetes. Conversely, they had lower HDL cholesterol levels and a slightly lower eGFR. Serum concentrations of all considered FAs increased progressively with increasing HOMA‐IR levels.

**TABLE 1:** Features of the study participants according to body mass index (BMI).

	BMI < 25 kg/m^2^ (*n* = 783)	BMI 25–29.9 kg/m^2^ (*n* = 769)	BMI ≥ 30 kg/m^2^ (*n* = 863)	*p*
Age (years)	42.7 (0.8)	48.8 (0.7)	46.7 (0.6)	< 0.001
BMI (kg/m^2^)	22.0 (0.1)	27.4 (0.1)	36.7 (0.3)	< 0.001
Male sex (%)	44.7 (2.2)	59.6 (1.5)	42.9 (1.5)	< 0.001
Race‐ethnicity (%)				
Non‐Hispanic White	66.3 (2.9)	66.9 (3.6)	65.3 (4.4)	< 0.001
Hispanic	11.6 (2)	15.7 (2.9)	17.1 (3.1)	
Non‐Hispanic Black	8.9 (1.4)	10.7 (1.4)	14.4 (2.3)	
Other	13.1 (1.6)	6.7 (1)	3.3 (0.6)	
SBP (mmHg)	117.0 (0.7)	119.5 (0.7)	124.6 (0.6)	< 0.001
Diabetes (%)	3.7 (0.7)	10.8 (1.1)	18.3 (1.3)	< 0.001
Triglycerides (mg/dL)	92.1 (2.7)	126.8 (5.3)	153.1 (5.0)	< 0.001
Total cholesterol (mg/dL)	182.6 (1.7)	191.2 (1.9)	192.2 (1.7)	< 0.001
HbA1c (%)	5.3 (0.0)	5.6 (0.0)	5.8 (0.0)	< 0.001
HDL (mg/dL)	61.5 (0.8)	52.3 (0.6)	48.6 (0.5)	< 0.001
AST (IU/L)	24.8 (0.8)	25.3 (1.0)	25.3 (0.8)	0.653
ALT (IU/L)	22.1 (0.9)	24.3 (0.6)	27.1 (0.7)	< 0.001
FIB4	1.1 (0.1)	1.2 (0.0)	1.0 (0.0)	0.152
FLI	14.9 (0.6)	44.8 (1.0)	82.7 (0.7)	< 0.001
eGFR (mL/min)	98.4 (0.9)	92.1 (0.8)	94.5 (0.9)	0.026
UACR (mg/g)	30.3 (8.2)	21.5 (3.9)	46.0 (9.7)	0.157
Saturated FAs				
Palmitic acid (16:0) (μmol/L)	2491.6 (33.9)	2812.6 (51.6)	3052.7 (54.2)	< 0.001
Stearic acid (18:0) (μmol/L)	600.5 (6.4)	652.7 (10.1)	693.9 (9.7)	< 0.001
MUFAs				
Palmitoleic acid (16:1n‐7) (μmol/L)	205.3 (5.5)	247.5 (9.4)	289.4 (7.7)	< 0.001
Oleic acid (18:1n‐9) (μmol/L)	1822.9 (30.9)	2109.8 (45.8)	2296.4 (51.1)	< 0.001
n‐6 PUFAs				
Linoleic acid (18:2n‐6) (μmol/L)	3228.8 (34.1)	3463.6 (41.4)	3554.7 (41.0)	< 0.001
Arachidonic acid (20:4n‐6) (μmol/L)	772.2 (9.9)	850.9 (11.5)	866.7 (11.1)	< 0.001
n‐3 PUFAs				
Alpha‐linolenic acid (18:3n‐3) (μmol/L)	73.4 (1.6)	84.6 (1.9)	92.1 (2.4)	< 0.001
Eicosapentaenoic acid (20:5n‐3) (μmol/L)	60.3 (2.1)	64.0 (2.3)	64.1 (2.5)	0.207
Docosahexaenoic acid (22:6n‐3) (μmol/L)	154.9 (3.6)	153.5 (3.4)	151.2 (3.2)	0.412
DHA/EPA ratio	3.2 (0.1)	2.9 (0.1)	2.9 (0.1)	0.020

*Note:* Data are represented as means (standard error) or percentage (standard error) according to the NHANES guidelines.

Abbreviations: ALT, alanine aminotransferase; AST, aspartate aminotransferase; BMI, body mass index; DHA, docosahexaenoic acid; eGFR, estimated glomerular filtration rate; EPA, eicosapentaenoic acid; FIB‐4, fibrosis 4 index; HbA1c, haemoglobin A1c; HDL, high density lipoprotein; HOMA‐IR, homeostatic model assessment of insulin resistance; SBP, systolic blood pressure; UACR, urinary albumin to creatinine ratio.

**TABLE 2:** Features of the study participants according to homeostatic model assessment‐insulin resistance (HOMA‐IR) quartiles.

	HOMA‐IR quartiles				
	1 (*n* = 608)	2 (*n* = 612)	3 (*n* = 613)	4 (*n* = 607)	Total
Age (years)	45.2 (0.8)	44.6 (0.8)	47.1 (0.8)	48.2 (0.8)	46.2 (0.4)
BMI (kg/m^2^)	24.4 (0.2)	27.0 (0.3)	30.2 (0.3)	35.6 (0.4)	29.1 (0.2)
Male sex (%)	47.1 (3.3)	48.3 (2.4)	49.6 (2.9)	50.5 (2.0)	48.8 (1.0)
Race‐ethnicity (%)					
Non‐Hispanic White	71.3 (3.5)	66.7 (3.3)	62.8 (4.1)	63.3 (4.7)	66.1 (3.5)
Hispanic	10.3 (2.2)	13.1 (2.4)	18 (2.8)	18.9 (3.9)	14.9 (2.5)
Non‐Hispanic Black	10.1 (1.5)	12.6 (1.8)	11.5 (2.0)	12 (2.5)	11.5 (1.5)
Other	8.4 (1.5)	7.7 (0.9)	7.7 (1.3)	5.8 (1.3)	7.4 (0.8)
SBP (mmHg)	117.0 (0.7)	118.6 (0.8)	121.2 (0.8)	126.2 (0.8)	120.6 (0.4)
Diabetes (%)	2.2 (0.6)	4.6 (0.9)	11 (1.4)	29.2 (2.3)	11.4 (0.7)
Significant alcohol consumption (%)	9.5 (1.2)	6.1 (1.1)	7.7 (2.2)	4.2 (1.4)	7 (0.8)
Triglycerides (mg/dL)	92.0 (5.4)	106.1 (3.6)	130.8 (4.4)	178.7 (6.5)	125.7 (2.7)
Total cholesterol (mg/dL)	187.5 (1.9)	187.5 (2.1)	190.7 (2.2)	190.4 (2.1)	189.0 (1.0)
HbA1c (%)	5.3 (0.0)	5.4 (0.0)	5.6 (0.0)	6.2 (0.1)	5.6 (0.0)
HDL (mg/dL)	62.4 (0.9)	55.4 (0.7)	51.1 (0.6)	45.2 (0.6)	53.8 (0.4)
AST (IU/L)	25.7 (1.3)	23.1 (0.6)	25.5 (1.3)	26.2 (0.5)	25.1 (0.5)
ALT (IU/L)	21.8 (0.8)	21.7 (0.5)	25.4 (1.0)	30.1 (0.8)	24.6 (0.4)
FIB4	1.2 (0.0)	1.0 (0.0)	1.2 (0.1)	1.1 (0.0)	1.1 (0.0)
FLI	23.5 (1.1)	38.8 (1.4)	58.4 (1.4)	81.0 (1.1)	49.4 (0.8)
eGFR (mL/min)	95.4 (1.0)	96.1 (1.0)	94.0 (1.1)	94.1 (1.1)	94.9 (0.5)
UACR (mg/g)	32.2 (9.3)	18.3 (2.7)	25.2 (5.8)	81.0 (26.8)	38.6 (7.1)
Saturated FAs					
Palmitic acid (16:0) (μmol/L)	2503.0 (45.0)	2582.9 (44.9)	2864.2 (51.5)	3299.5 (74.5)	2801.6 (28.6)
Stearic acid (18:0) (μmol/L)	604.3 (9.7)	617.4 (9.0)	666.4 (10.0)	724.4 (12.5)	651.5 (5.3)
MUFAs					
Palmitoleic acid (16:1n‐7) (μmol/L)	216.0 (8.9)	215.2 (7.7)	257.1 (8.9)	314.8 (9.8)	249.5 (4.6)
Oleic acid (18:1n‐9) (μmol/L)	1826.4 (41.5)	1894.2 (38.4)	2140.5 (47.3)	2534.9 (69.7)	2089.4 (26.2)
n‐6 PUFAs					
Linoleic acid (18:2n‐6) (μmol/L)	3221.7 (39.8)	3369.6 (41.6)	3490.8 (45.1)	3647.2 (56.3)	3426.0 (23.3)
Arachidonic acid (20:4n‐6) (μmol/L)	796.5 (11.2)	822.5 (12.8)	848.7 (13.4)	866.0 (13.3)	832.4 (6.4)
n‐3 PUFAs					
Alpha‐linolenic acid (18:3n‐3) (μmol/L)	69.2 (1.6)	79.9 (2.2)	87.9 (2.5)	100.1 (2.9)	83.8 (1.2)
Eicosapentaenoic acid (20:5n‐3) (μmol/L)	59.4 (2.4)	59.7 (2.0)	63.6 (2.4)	68.6 (3.7)	62.7 (1.3)
Docosahexaenoic acid (22:6n‐3) (μmol/L)	148.1 (3.6)	151.6 (3.5)	155.0 (3.9)	157.7 (4.6)	153.0 (2.0)
DHA/EPA ratio	3.0 (0.1)	3.1 (0.1)	3.0 (0.1)	2.9 (0.1)	3.0 (0.0)

*Note:* Data are represented as means (standard error) or percentage (standard error) according to the NHANES guidelines.

Abbreviations: ALT, alanine aminotransferase; AST, aspartate aminotransferase; BMI, body mass index; DHA, docosahexaenoic acid; eGFR, estimated glomerular filtration rate; EPA, eicosapentaenoic acid; FIB‐4, fibrosis 4 index; HbA1c, haemoglobin A1c; HDL, high density lipoprotein; HOMA‐IR, homeostatic model assessment of insulin resistance; SBP, systolic blood pressure; UACR, urinary albumin to creatinine ratio.

### 3.1 Characteristics of the Study Population According to Steatosis and Fibrosis

Liver steatosis (defined as a FLI ≥ 60) was present in 41.8% (95% CI 38.9–44.8) of all considered participants. Features of participants according to liver steatosis are shown in Table S1. Participants with steatosis were more commonly men, showed higher BMI, SBP, triglycerides, HbA1c, AST, ALT, HOMA‐IR and UACR; they also had a higher prevalence of diabetes. Conversely, they had lower HDL cholesterol levels and a slightly lower eGFR. Serum concentrations of all considered FAs were significantly higher among participants with steatosis.

Liver fibrosis (defined as a FIB‐4 ≥ 1.3) was present in 28.4% (95% CI 26.6–30.2) of all considered participants. Participants with fibrosis were significantly older and more commonly men, showed higher SBP and HbA1c levels and had a higher prevalence of diabetes (Table S2). Conversely, they had lower eGFR levels. Serum concentrations of all considered FAs did not differ significantly between participants with and without fibrosis.

### 3.2 Association of Circulating FAs With Insulin Resistance, Liver Steatosis and Fibrosis

Tables 3 and 4 report the results of logistic regression analyses on the association between FAs expressed as a percentage of total FA levels and HOMA‐IR and FLI and FIB‐4, respectively. The binary dependent variables were HOMA‐IR ≥ 2.5, FLI ≥ 60 and FIB‐4 ≥ 1.3. All analyses were adjusted for age, sex, BMI and race‐ethnicity. FA levels were log‐transformed. As shown in Table 3, higher levels of palmitic, palmitoleic, oleic and alpha‐linolenic acid were significantly associated with insulin resistance. Conversely, linoleic acid, arachidonic acid and DHA were inversely associated with insulin resistance.

**TABLE 3:** Logistic regression model assessing the association between fatty acids expressed as percentage of total fatty acids and estimated insulin resistance after adjustment for confounders.

Outcome: HOMA‐IR > 2.5	OR	95% CI	*p*
Saturated FAs			
Palmitic acid (16:0)	1.23	1.12–1.35	< 0.001
Stearic acid (18:0)	0.93	0.83–1.05	0.228
MUFAs			
Palmitoleic acid (16:1n‐7)	1.38	1.14–1.67	0.003
Oleic acid (18:1n‐9)	1.16	1.10–1.23	< 0.001
n‐6 PUFAs			
Linoleic acid (18:2n‐6)	0.92	0.89–0.95	< 0.001
Arachidonic acid (20:4n‐6)	0.82	0.76–0.88	< 0.001
n‐3 PUFAs			
alpha‐Linolenic acid (18:3n‐3)	2.72	1.38–5.36	0.007
Eicosapentaenoic acid (20:5n‐3)	0.85	0.69–1.05	0.119
Docosahexaenoic acid (22:6n‐3)	0.60	0.51–0.70	< 0.001
DHA/EPA ratio	0.96	0.90,1.03	0.259

*Note:* Results were adjusted for age, sex, BMI, race‐ethnicity. Age and BMI were included as continuous variables. Fatty acid concentrations were log‐transformed; therefore the reported odds ratios (ORs) represent the increase in odds of a HOMA‐IR > 2.5 for each increase in unit of log‐FA.

Abbreviations: DHA, docosahexaenoic acid; EPA, eicosapentaenoic acid; FAs, fatty acids; HOMA‐IR, homeostatic model assessment of insulin resistance; MUFA, monounsaturated fatty acids; PUFAs, polyunsaturated fatty acids.

**TABLE 4:** Logistic regression model assessing the association between fatty acids expressed as percentage of total fatty acids and both liver steatosis (evaluated through the fatty liver index) and liver fibrosis (evaluated through the fibrosis‐4 index) in included participants.

Variable	FLI > 60			FIB‐4 > 1.3		
	OR	95% CI	*p*	OR	95% CI	*p*
Saturated FAs						
Palmitic acid (16:0) (%)	1.37	1.26–1.50	< 0.001	1.04	0.97–1.11	0.268
Stearic acid (18:0) (%)	0.83	0.68–1.02	0.070	1.40	1.09–1.81	0.013
MUFAs						
Palmitoleic acid (16:1n‐7) (%)	2.11	1.61–2.75	< 0.001	1.15	0.92–1.43	0.196
Oleic acid (18:1n‐9) (%)	1.24	1.19–1.30	< 0.001	0.97	0.92–1.02	0.203
n‐6 PUFAs						
Linoleic acid (18:2n‐6) (%)	0.87	0.83–0.92	< 0.001	0.98	0.95–1.01	0.134
Arachidonic acid (20:4n‐6) (%)	0.76	0.72–0.81	< 0.001	1.02	0.92–1.15	0.656
n‐3 PUFAs						
Alpha‐Linolenic acid (18:3n‐3) (%)	3.27	1.98–5.39	< 0.001	0.48	0.25–0.93	0.031
Eicosapentaenoic acid (20:5n‐3) (%)	0.72	0.53–0.96	0.028	1.17	0.85–1.61	0.317
Docosahexaenoic acid (22:6n‐3) (%)	0.49	0.41–0.58	< 0.001	1.24	1.05–1.48	0.016

*Note:* Results were adjusted for age, sex, BMI, race‐ethnicity. Age and BMI were included as continuous variables. Fatty acid concentrations were log‐transformed; therefore the reported ORs represent the increase in odds of liver steatosis and fibrosis for each increase in unit of log‐FA.

Abbreviations: DHA, docosahexaenoic acid; EPA, eicosapentaenoic acid; FAs, fatty acids; FIB‐4, fibrosis‐4 index; FLI, fatty liver index; MUFA, monounsaturated fatty acids; PUFAs, polyunsaturated fatty acids.

As shown in Table 4, palmitic, palmitoleic, oleic and alpha‐linolenic acid were directly associated with elevated FLI, while an indirect relationship was found with linoleic acid, arachidonic acid EPA and DHA. Finally, while stearic acid was associated with an increased risk of elevated FIB‐4, an opposite association was found for alpha‐linolenic acid. Results are also reported in graphical form (forest plots) in Figures S1–S3.

## 4 Discussion

In this large population‐based cross‐sectional study conducted in the large NHANES database, we evaluated the impact of circulating FAs levels on the risk of insulin resistance, liver steatosis and liver fibrosis. We made a series of observations. First, most circulating FAs levels were significantly and directly associated with a higher risk of insulin resistance and MASLD when evaluated in absolute terms. Nonetheless, specific associations were found between different FA classes and liver outcomes when the latter were evaluated as a percentage of total FAs. Second, while no evidence of an association with most FAs and liver fibrosis was identified, alpha‐linolenic acid was associated with a lower risk of elevated FIB4 (> 1.3). Third, the DHA/EPA ratio, which is considered to be a marker of PPAR‐α activation, was inversely associated with the risk of steatosis. These findings were achieved using non‐invasive serum‐based biomarkers of liver steatosis and fibrosis and by measuring total FA concentrations by gas chromatography–mass spectrometry and by applying multivariable logistic regression models.

The circulating FAs levels, when expressed as absolute concentrations, were associated with the estimated insulin resistance (HOMA‐IR) regardless of the degree of saturation (saturated, MUFA and PUFA), and regardless of whether they were n‐3 or n‐6 long chain PUFAs. Similarly, the absolute circulating FAs level was associated with higher FLI and also in this case the relationship was detectable regardless of the degree of saturation and regardless of whether n‐3 or n‐6 long chain PUFA. A potential explanation for these associative findings is that the absolute circulating FAs level might be influenced by the total dietary intake. To further corroborate this hypothesis, it can be noted that the circulating levels of the essential FAs linoleic and alpha‐linolenic acids, which cannot be synthesised and are determined by the dietary intake only, are strongly associated with HOMA‐IR and FLI. Nonetheless, when expressed as a percentage of total circulating FAs, we showed that PUFAs (and in particular linoleic, arachidonic, DHA and EPA) were inversely associated with liver steatosis. In this sense a diet composition that is richer in PUFAs compared with saturated fats might be beneficial for MASLD.

In contrast with the homogeneous association of the circulating FAs level with HOMA‐IR and FLI, the circulating level of most FAs was not associated with a higher risk of liver fibrosis (FIB‐4 > 1.3), except for stearic acid. On the other hand, the notable protective exception of alpha‐linolenic acid was identified. The lack of a robust association with the surrogate biomarker of fibrosis may not be surprising because this kind of organ damage at the liver site may require a relatively long period of time to take place while the level of the FAs may be changing rather quickly, within a shorter period of time, making it unlikely to find an association between variables with different temporal manifestations. On the other hand, the inverse association with alpha‐linolenic acid is quite intriguing because it has been a matter of discussion whether the dietary shift of Western diets towards the n‐6 long chain PUFAs, with high content in cereals, eggs, animal fat, whole grain breads, sunflower and corn oils, at the expense of n‐3 long chain PUFAs abundant in leafy green vegetables, walnuts and canola, flaxseeds and rapeseed oils (alpha‐linoleic acid) or in marine foods (EPA or DHA) may be detrimental for liver health [38]. Mechanistic explanations are related to the ability of n‐3 long chain PUFAs to downregulate the transcription factor sterol regulatory element binding protein‐1 (SREBP‐1) [39, 40], which is a robust promoter of triglyceride accumulation in the liver [41]; evidence shows that they are also able to up‐regulate peroxisomal proliferator activated receptor‐alpha [40] which stimulates fatty acid oxidation and increases transcription of fatty acid degradation genes mitochondrial carnitine palmitoyl transferase‐1 (CPT‐1) [42]. More recently, it was shown that n‐3 long chain PUFAs decreased the expression of genes that promote fibrosis in both activated liver cells responsible for scarring and in the liver affected by fibrosis [43].

In humans, we have observational findings. It was described that individuals with NAFLD had reduced hepatic content of n‐3 long chain PUFAs and an abnormally high n‐6/n‐3 PUFAs ratio [44, 45]. An analysis of the UK Biobank showed that the regular consumption of n‐3 long chain PUFAs was associated with the reduction of the onset of hepatic diseases [46].

More controversy is present when interventional studies are taken into consideration.

Initial randomised studies showed beneficial effects of n‐3 PUFA supplementation in NAFLD in terms of reduction in transaminase levels and liver fat content [47, 48].

In a study in which oral supplementation of fish oil capsules with 503 mg docosahexaenoic acid (DHA) and 103 mg eicosapentaenoic acid (EPA) three times daily for 6 months showed a significant reduction of controlled attenuation parameter and of liver stiffness measurement using Fibroscan [49]. Similar results during a similar observational period were observed using a supply of 50 mL of a solution of n‐3 PUFA in a 1:1 ratio of EHA and DHA [50]. A positive finding was reported with n‐3 supplementation at a dose of 3000 mg/day for 1 year in patients with MASH with a reduction in liver fat, independent of body weight loss but no significant changes in histological activity [51]. This confirmed the findings of a previous study performed with 4 g/day of DHA + EPA [52]. By contrast, Sanyal et al. [53] assessed various doses of EPA supplementation in patients with MASLD and MASH, but only the high doses (2700 mg/day) reduced the serum TG levels, with no effect on steatosis, inflammation, ballooning or fibrosis. A potential explanation for these controversial findings may be related to the different doses and composition of the tested n‐3 long chain PUFAs. Interestingly not only the composition of the two categories of n‐6 compared with n‐3 long chain PUFAs but also the composition within the same category of n‐3 long chain PUFAs, may be influencing the outcome as suggested in a study in humans in which the treatment with DHA was more effective than the intervention with EPA in modulating biomarkers of inflammation [54].

In line with this observation, the logistic regression model performed in our analysis detected an interesting inverse association between the DHA/EPA ratio and the surrogate biomarker of liver steatosis. This result is still exploratory and needs to be validated in future studies. Indeed a recent meta‐analysis showed that DHA increased total cholesterol and insulin levels compared with EPA [55]. It is not straightforward to deduce how the present study might inform future clinical trials in humans. When liver steatosis is considered as the primary outcome (e.g., through MRI‐PDFF) a diet enriched in linoleic, arachidonic and DHA might be more beneficial. On the other hand, if fibrosis regression is to be achieved, our study suggests the use of alpha‐linolenic acid as the molecule of choice. In dietary terms, the use of flaxseeds and flaxseed oil, walnuts and walnut oil, chia seeds, and soybeans and soybean oil, which contain the highest amounts of this FA, should be promoted.

This study has several strengths. Being based on data obtained from NHANES survey cycles, it provides representative data that can be generalised to the entire US multiethnic adult population. Moreover, collection of both biochemical and risk factor data was performed in a standardised and homogenous fashion by trained personnel. Finally, methods used to measure FAs are characterised by high sensitivity and specificity.

On the same lines, several limitations should also be acknowledged. First, the cross‐sectional design of this study makes it impossible to draw conclusions on the cause‐effect relation between FAs and insulin resistance/MASLD. Second, the absence of liver histologic data or imaging data prevents us from reporting the exact prevalence of steatosis and significant fibrosis according to the gold standard technique. It should be stressed, however, that liver biopsy is a time‐consuming, invasive and costly procedure not well suited for large epidemiologic studies. While the use of FLI to diagnose liver steatosis is not recommended in clinical practice, guidelines allow its use in large epidemiologic studies such as the present. Moreover, since its first description it has been validated in numerous populations showing good accuracy compared with either liver ultrasound or magnetic resonance spectroscopy [56]. On the other hand, the use of FIB‐4 is recommended by guidelines as the first step in the identification of patients with possible advanced liver fibrosis. While we adjusted for known risk factors for MASLD and liver fibrosis, we could not adjust for all potential confounders including physical exercise. Finally, while statistical power was sufficient to perform analyses within the entire population, it was not enough to conduct meaningful subgroup analysis according to ethnicity or dyslipidaemia. Larger studies might account for these shortcomings.

In conclusion, this large cross‐sectional study shows that circulating FA levels are associated with insulin resistance and steatosis, but it also provides initial epidemiologic evidence in humans that ω‐3 PUFA are associated with a lower risk of liver steatosis and fibrosis. Moreover, manipulation of their circulating levels using nutritional strategies or supplementation with different compositions deserves to be tested in clinical studies.

## Author Contributions

S.C. and G.P. designed the study, wrote, reviewed and edited the manuscript. S.C. researched and analysed data. All authors reviewed and edited the manuscript. All authors approved the final version of the manuscript to be published. S.C. is the guarantor of this work.

## Disclosure

Writing Assistance: The manuscript was written by the authors without any external writing assistance.

## Conflicts of Interest

The authors declare no conflicts of interest.

## Supporting information


**Appendix S1:** liv70441‐sup‐0001‐AppendixS1.pdf.

## Data Availability

All data used in this study are available publicly at https://wwwn.cdc.gov/nchs/nhanes/Default.aspx.
