# Supplementary material for: Qualitative Properties of Circulating Fatty Acids Are Associated With MASLD: A Cross‐Sectional Study From the NHANES Database
Source: Liver Int. 2025 Nov 14;45(12):e70441. doi: 10.1111/liv.70441 (PMC12617388; doi:10.1111/liv.70441)
Supplement: Supplementary file 1 — Appendix S1: liv70441‐sup‐0001‐AppendixS1.pdf. [file LIV-45-0-s001.pdf]

Supplementary Table 1 Features of the included participants according to fatty liver index (FLI) values.

	Fatty Liver Index			
	<60		≥60	
Age (years)	44.1	(0.6)	48.5	(0.6)
BMI (Kg/m2)	24.8	(0.1)	35.1	(0.3)
Male sex (%)	47.3	(1.7)	51.9	(1.8)
Race-ethnicity (%)				
Non-Hispanic White	65.2	(3.1)	66.9	(4.4)
Hispanic	13.6	(2.4)	17.3	(3.3)
Non-Hispanic Black	11.2	(1.3)	12.1	(2.2)
Other	10	(1.2)	3.7	(0.7)
SBP (mmHg)	117.3	(0.5)	125.2	(0.6)
Diabetes (%)	5.3	(0.8)	19	(1.2)
Significant alcohol consumption (%)	6.3	(0.8)	8.5	(1.3)
Triglycerides (mg/dL)	89.1	(1.6)	176.4	(5.6)
Cholesterol, total (mg/dL)	183.9	(1.3)	196.2	(1.7)
HbA1c (%)	5.4	(0.0)	5.9	(0.0)
Direct HDL-Cholesterol (mg/dL)	58.7	(0.6)	46.8	(0.5)
AST (IU/L)	23.5	(0.5)	27.3	(1.0)
ALT (IU/L)	21.4	(0.5)	29.3	(0.7)
FIB4	1.1	(0.0)	1.1	(0.0)
HOMA-IR	1.9	(0.1)	5.7	(0.3)
eGFR (ml/min)	96.9	(0.7)	92.9	(0.8)
UACR (mg/g)	24.0	(4.6)	46.5	(9.1)
Saturated FAs				
Palmitic acid (16:0) (umol/L)	2444.1	(23.0)	3291.0	(55.9)
Stearic acid (18:0) (umol/L)	593.3	(4.7)	733.5	(10.4)
MUFAs				
Palmitoleic acid (16:1n-7) (umol/L)	194.1	(3.7)	326.1	(9.0)
Oleic acid (18:1n-9) (umol/L)	1774.7	(19.5)	2521.6	(52.9)
n-6 PUFAs				
Linoleic acid (18:2n-6) (umol/L)	3246.0	(25.9)	3670.6	(40.7)
Arachidonic acid (20:4n-6) (umol/L)	794.0	(7.8)	883.8	(10.8)
n-3 PUFAs				
alpha-Linolenic acid (18:3n-3) (umol/L)	72.0	(1.1)	100.2	(2.4)
Eicosapentaenoic acid (20:5n-3) (umol/L)	59.0	(1.5)	68.7	(2.5)
Docosahexaenoic acid (22:6n-3) (umol/L)	149.2	(2.5)	158.2	(3.4)
DHA/EPA ratio	3.1	(0.0)	2.8	(0.0)

Data are represented as means (standard error) or percentage (standard error) according to the NHANES guidelines.

Abbreviations: BMI, body mass index; SBP, systolic blood pressure; HbA1c, hemoglobin A1c; HDL, high density lipoprotein; AST, aspartate aminotransferase; ALT, alanine aminotransferase; FIB-4, Fibrosis 4 index; HOMA-IR, homeostatic model assessment of

insulin resistance; eGFR, estimated glomerular filtration rate; UACR, urinary albumin to creatinine ratio; DHA, docosahexaenoic acid; EPA, eicosapentaenoic acid.

Supplementary Table 2 Features of the included participants according to Fibrosis-4 (FIB-4) values.

	Fibrosis-4			
	<1.3		≥1.3	
Age (years)	39.1	(0.4)	64.1	(0.5)
BMI (Kg/m2)	29.3	(0.2)	28.6	(0.3)
Male sex (%)	46.2	(1.4)	55.5	(1.8)
Race-ethnicity (%)				
Non-Hispanic White	62.3	(3.5)	75.8	(3.5)
Hispanic	17.9	(2.7)	7.5	(1.9)
Non-Hispanic Black	12.2	(1.6)	9.7	(1.6)
Other	7.6	(0.8)	7	(1.1)
SBP (mmHg)	117.6	(0.4)	128.0	(0.9)
Diabetes (%)	8.9	(0.9)	17.8	(1.1)
Significant alcohol consumption (%)	6.2	(0.9)	9	(1.2)
Triglycerides (mg/dL)	124.5	(3.4)	128.5	(4.3)
Cholesterol, total (mg/dL)	188.5	(1.2)	189.9	(2.0)
HbA1c (%)	5.5	(0.0)	5.9	(0.1)
Direct HDL-Cholesterol (mg/dL)	52.8	(0.4)	56.3	(0.8)
AST (IU/L)	22.5	(0.2)	31.8	(1.6)
ALT (IU/L)	23.7	(0.4)	27.1	(1.1)
FLI	48.4	(1.0)	52.0	(1.4)
HOMA-IR	3.4	(0.2)	3.6	(0.3)
eGFR (ml/min)	101.7	(0.6)	78.0	(0.9)
UACR (mg/g)	29.7	(5.1)	61.5	(21.4)
Saturated FAs				
Palmitic acid (16:0) (umol/L)	2778.9	(34.0)	2855.0	(53.3)
Stearic acid (18:0) (umol/L)	645.4	(6.6)	665.9	(8.7)
MUFAs				
Palmitoleic acid (16:1n-7) (umol/L)	244.2	(5.1)	262.1	(9.5)
Oleic acid (18:1n-9) (umol/L)	2050.8	(31.8)	2183.9	(45.3)
n-6 PUFAs				
Linoleic acid (18:2n-6) (umol/L)	3445.8	(28.0)	3371.7	(41.5)
Arachidonic acid (20:4n-6) (umol/L)	818.5	(7.5)	866.1	(11.9)
n-3 PUFAs				
alpha-Linolenic acid (18:3n-3) (umol/L)	83.9	(1.5)	83.4	(2.0)
Eicosapentaenoic acid (20:5n-3) (umol/L)	56.4	(1.3)	78.6	(3.3)
Docosahexaenoic acid (22:6n-3) (umol/L)	142.8	(2.1)	178.1	(4.3)
DHA/EPA ratio	3.1	(0.0)	2.8	(0.1)

Data are represented as means (standard error) or percentage (standard error) according to the NHANES guidelines.

Abbreviations: BMI, body mass index; SBP, systolic blood pressure; HbA1c, hemoglobin A1c; HDL, high density lipoprotein; AST, aspartate aminotransferase; ALT, alanine

aminotransferase; FIB-4, Fibrosis 4 index; HOMA-IR, homeostatic model assessment of insulin resistance; eGFR, estimated glomerular filtration rate; UACR, urinary albumin to creatinine ratio; DHA, docosahexaenoic acid; EPA, eicosapentaenoic acid.

Figure S1 Forest plot showing the adjusted OR (95% CI) of fatty acids associated with insulin resistance.

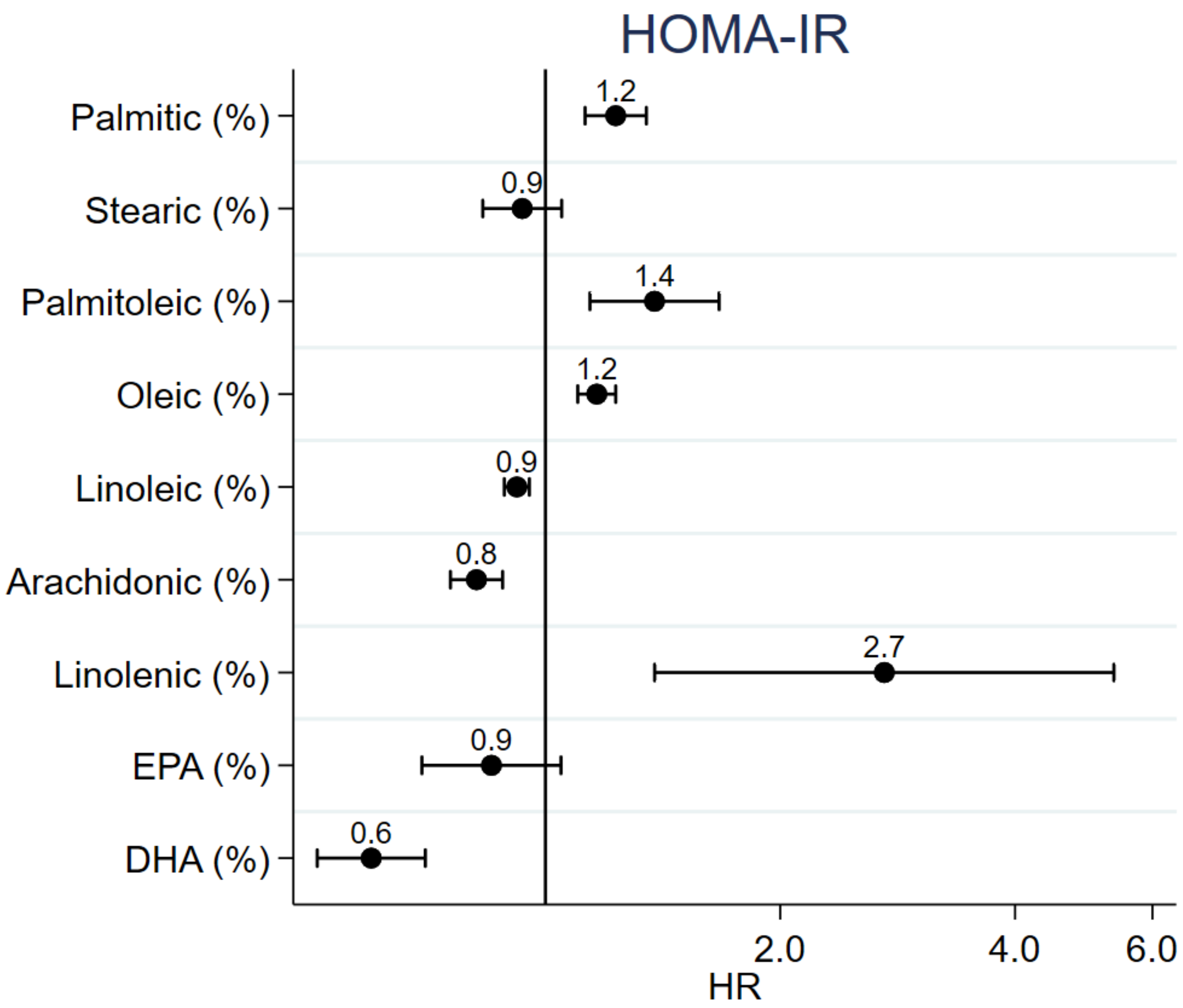

Results were adjusted for age, sex, BMI, race-ethnicity. Age and BMI were included as continuous variables. Fatty acid concentrations were log-transformed; therefore the reported ORs represent the increase in Odds of liver steatosis and fibrosis for each increase in unit of log-FA.

Figure S2 Forest plot showing the adjusted OR (95% CI) of fatty acids associated with elevated FLI.

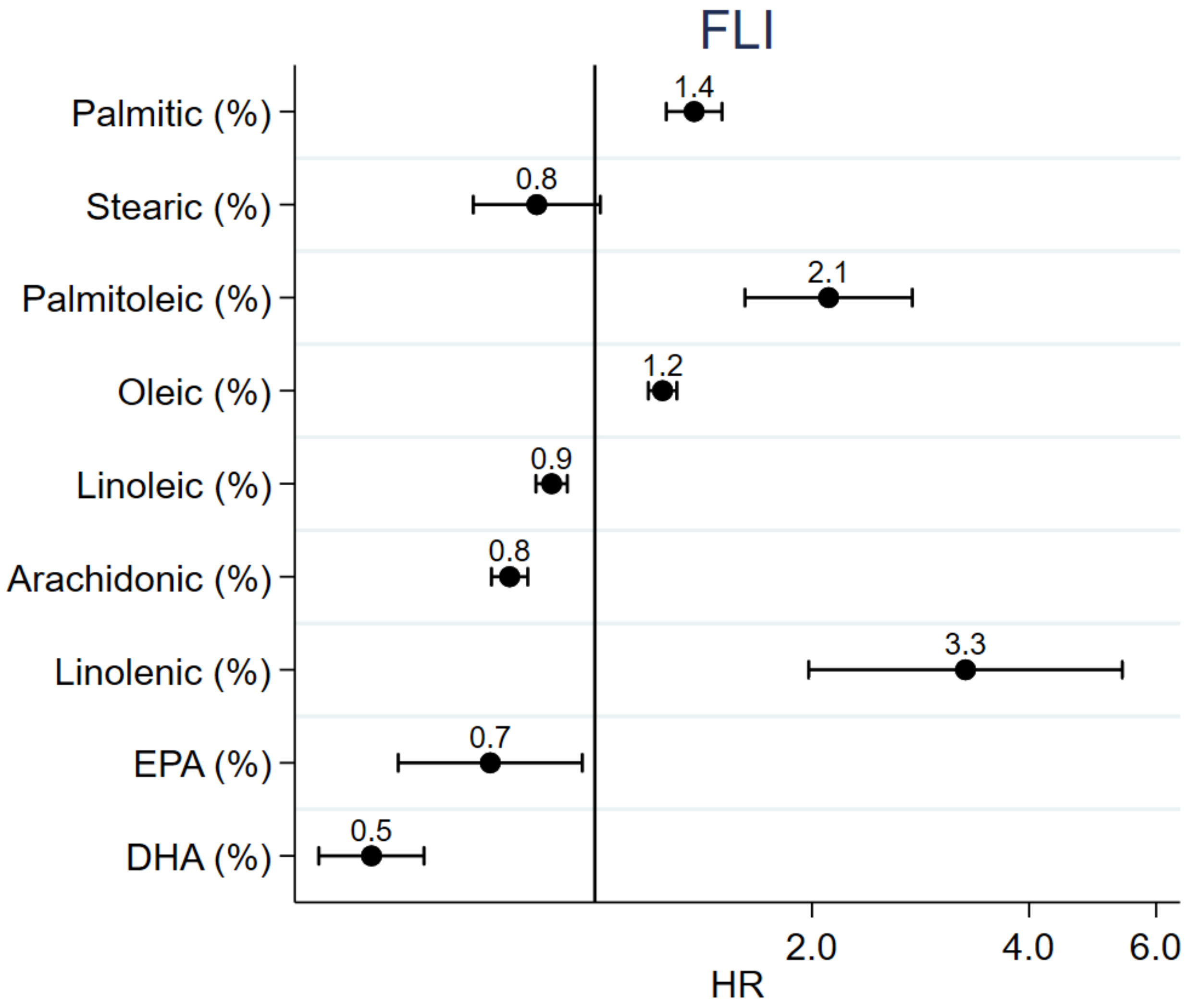

Results were adjusted for age, sex, BMI, race-ethnicity. Age and BMI were included as continuous variables. Fatty acid concentrations were log-transformed; therefore the reported ORs represent the increase in Odds of liver steatosis and fibrosis for each increase in unit of log-FA.

Figure S3 Forest plot showing the adjusted OR (95% CI) of fatty acids associated with elevated FLI.

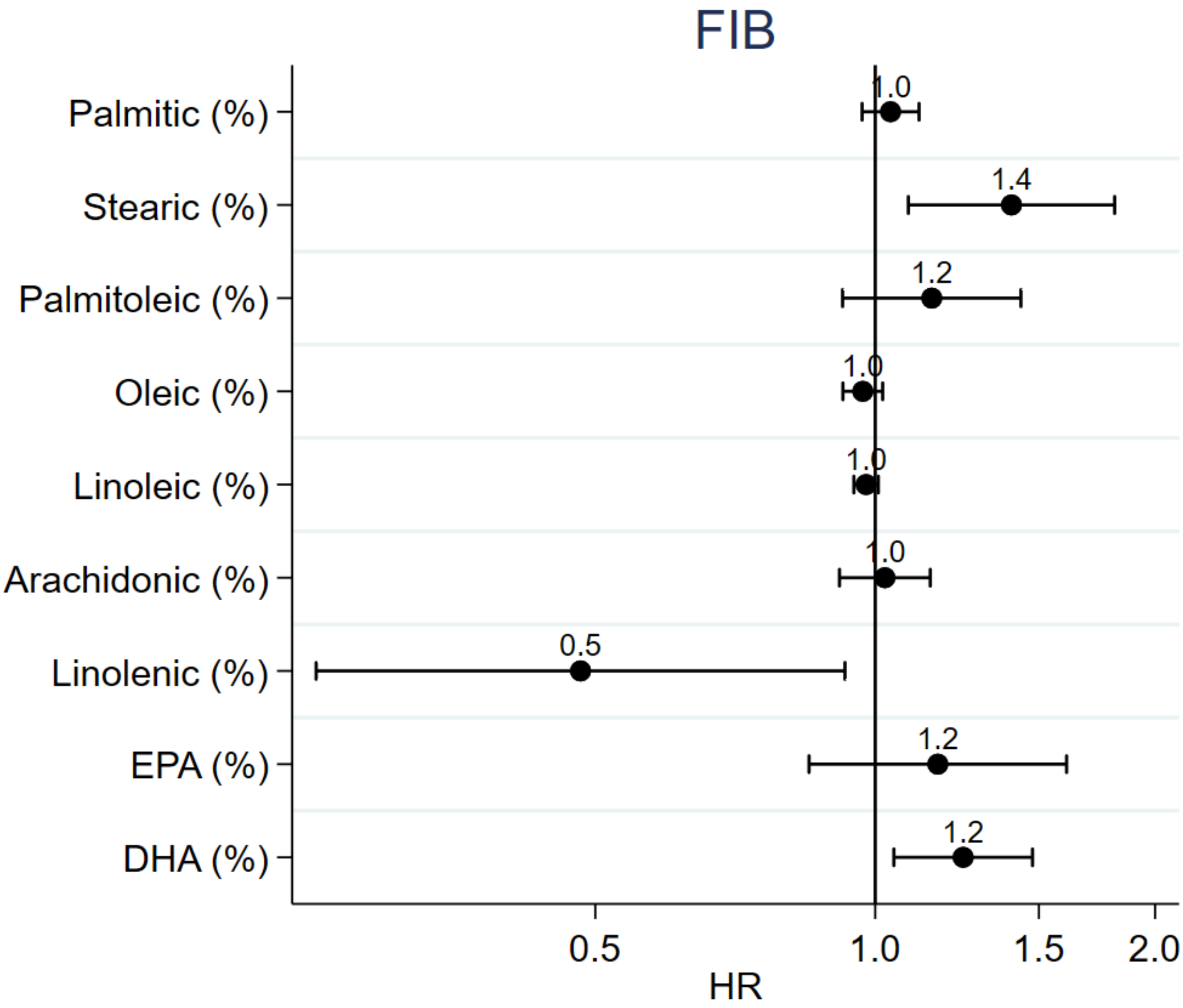

Results were adjusted for age, sex, BMI, race-ethnicity. Age and BMI were included as continuous variables. Fatty acid concentrations were log-transformed; therefore the reported ORs represent the increase in Odds of liver steatosis and fibrosis for each increase in unit of log-FA.
